# Micromanagement of Developmental and Stress-Induced Senescence: The Emerging Role of MicroRNAs

**DOI:** 10.3390/genes10030210

**Published:** 2019-03-12

**Authors:** Aleksandra Swida-Barteczka, Zofia Szweykowska-Kulinska

**Affiliations:** Department of Gene Expression, Institute of Molecular Biology and Biotechnology, Faculty of Biology, Adam Mickiewicz University, Poznań, Umultowska 89, 61-614 Poznan, Poland; zofszwey@amu.edu.pl

**Keywords:** senescence, microRNAs, plants, drought

## Abstract

MicroRNAs are short (19–24-nucleotide-long), non-coding RNA molecules. They downregulate gene expression by triggering the cleavage or translational inhibition of complementary mRNAs. Senescence is a stage of development following growth completion and is dependent on the expression of specific genes. MicroRNAs control the gene expression responsible for plant competence to answer senescence signals. Therefore, they coordinate the juvenile-to-adult phase transition of the whole plant, the growth and senescence phase of each leaf, age-related cellular structure changes during vessel formation, and remobilization of resources occurring during senescence. MicroRNAs are also engaged in the ripening and postharvest senescence of agronomically important fruits. Moreover, the hormonal regulation of senescence requires microRNA contribution. Environmental cues, such as darkness or drought, induce senescence-like processes in which microRNAs also play regulatory roles. In this review, we discuss recent findings concerning the role of microRNAs in the senescence of various plant species.

## 1. Introduction

MicroRNAs are 19–24-nucleotide-long, small, non-coding, single-stranded RNAs. MicroRNAs act at the post-transcriptional level, targeting specific mRNAs to trigger cleavage or translation inhibition. The degree of complementarity between a microRNA and its target mRNA determines whether the mRNA will be cleaved or its translation will be inhibited [1,2,3,4,5]. Founding microRNAs, first discovered in *Ceanorhabditis elegans*, are lin-4 and let-7, which are temporally expressed and govern larval phasing and larval-to-adult transitions [6,7,8]. MicroRNAs and their function in gene expression have been further identified in many eukaryotic species, as well as in plants. *Arabidopsis thaliana* was the first plant specimen in which microRNAs were identified. The number of different microRNAs varies between plant species, and for *A. thaliana* and *Oryza sativa* it is 428 and 738, respectively [9,10]. The degree of microRNA conservation ranges from those conserved within the whole *Viridiplantae* clade to non-conserved species-specific molecules. The unicellular algae *Chlamydomonas reinhardtii* is to some extent exceptional, because the vast majority of its identified microRNAs are specific to algae, and only three microRNA species are also found in liverworts [11,12].

### 1.1. Biogenesis and General Roles of Plant microRNAs

**Figure 1:**
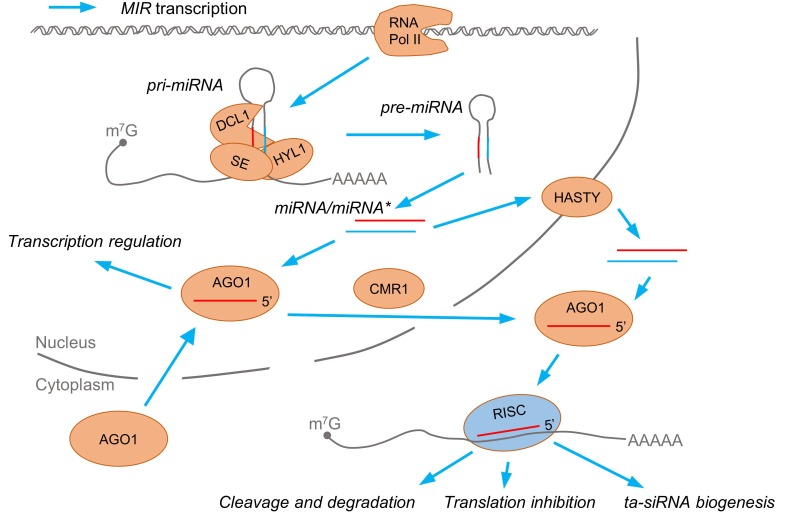
Biogenesis and functions of microRNAs in plants. MicroRNA genes (*MIR*) are transcribed by RNA polymerase II (RNA Pol II) to primary transcripts of microRNA (pri-miRNA) precursors. Pri-miRNAs are cleaved by a microprocessor complex with a core of DICER-LIKE1 (DCL1), HYPONASTIC LEAVES1 (HYL1), and SERRATE (SE). The intermediate steps of microRNA biogenesis are pre-miRNA forming a hairpin structure and the microRNA/microRNA* duplex. MicroRNA bound to AGO1 is exported from the nucleus in a CMR1-dependent way, while HASTY drives microRNA/microRNA* duplex export. MicroRNA directs AGO1 to play its roles in mRNA cleavage or translation inhibition, trans-acting short-interfering RNA (ta-siRNA) biogenesis, and transcription regulation.

MicroRNAs originate from genes that are hundreds to thousands of nucleotides long (*MIR*), which, in the case of plants, represent mostly independent transcriptional units. *MIRs* are transcribed by RNA polymerase II (RNA Pol II), and primary transcripts of microRNAs (pri-miRNAs) contain a 5’-cap and 3’-polyA tail (Figure 1) [13]. MicroRNA and its imperfectly paired partner, microRNA*, occupy a stem of a stem-loop structure (pre-miRNA) located in pri-miRNA. In plants, the trimming of pri-miRNA hairpins and the dicing out of the microRNA/microRNA* duplex is processed by RNase III enzyme DICER-LIKE1 (DCL1) [14,15]. DCL1, together with a dsRNA binding protein HYPONASTIC LEAVES1 (HYL1) and a zinc-finger-containing protein SERRATE (SE), forms a core of the microprocessor complex that produces miRNA/miRNA* duplexes. Many other proteins interact with DCL1, HYL1, or SE for proper microRNA biogenesis [16,17,18,19]. Then, mature microRNA is loaded into AGO1 and exported to the cytoplasm as an AGO1/microRNA complex with the assistance of CHROMOSOMAL REGION MAINTENANCE1 (CMR1/EXPORTIN1) [20]. Guide-strand selection from microRNA/microRNA* duplexes is directed in the nucleus by HYL1 [21]. It has also been shown that microRNA can be exported from the nucleus in a duplex with microRNA*—a process that is controlled by HASTY, an ortholog of exportin5 [22]. The microRNA* strand is usually degraded. In the cytosol, AGO1 loaded with microRNA is part of the RNA-induced silencing complex (RISC) and post-transcriptionally inhibits target mRNAs or sets phasing in trans-acting siRNA precursor processing [23]. Target mRNA expression is downregulated primarily by cleavage, while co-translational inhibition occurs less frequently [1,5,24,25]. AGO1 binding stabilizes microRNAs in the cytoplasm, while their expression is co-transcriptionally decreased by AGO1/microRNA action in the nucleus. This mechanism was shown for several salt stress-induced microRNAs [26]. The function of trans-acting short-interfering RNAs (ta-siRNAs) is similar to those maintained by microRNAs [27]. Twenty-one-nucleotide-long ta-siRNAs guide RISC to cleave target mRNAs. Ta-siRNAs, unlike ssRNA-originating microRNAs, are cleaved from dsRNA synthesized by RNA-dependent RNA polymerase 6 (RDR6) using RNA Pol II product as a template [23]. The dsRNA is a substrate for DCL-dependent sequential cleavage generating ta-siRNA duplexes.

### 1.2. Addressing Plant Senescence as a Part of the Developmental Process Has Many Positive Aspects

**Figure 2:**
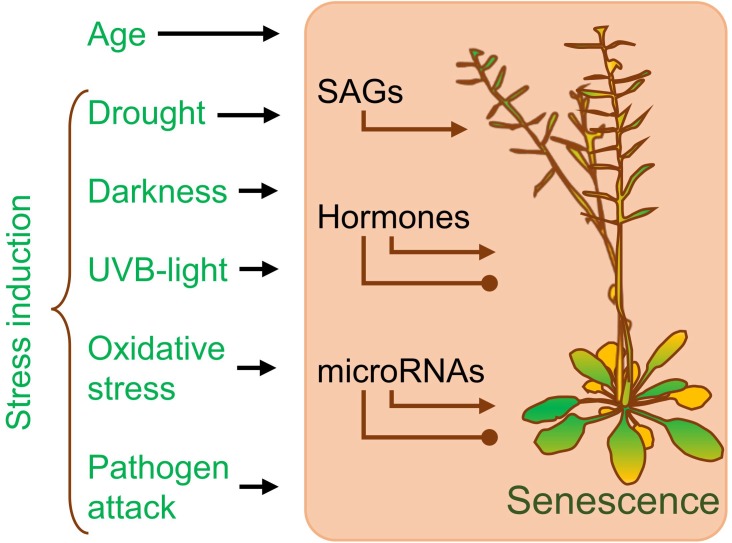
Senescence of a plant. Plant senescence is an age-related process influenced by various environmental factors. Senescence is regulated by hormones, microRNAs, and senescence-associated genes (SAGs). The arrows with triangular points reflect activation, and the arrows with circular tips reflect inhibition. UVB: ultraviolet B.

Plant life can be divided into two main phases: juvenile‒vegetative and mature‒reproductive. A mature plant is generally recognized as possessing reproductive organs. The morphology of the leaves and vegetative organs also distinguishes mature from juvenile plants. Senescence of a plant is an integral part of regular development. It leads to the death of a cell, organ, or whole plant. The process starts as an answer to internal signals, such as hormones and growth-regulating factors. The competence to answer senescence signals develops during the transition from the juvenile to the mature phase. Such fitness is linked to plant age and ability to reproduce. Therefore, only plants that have completed growth activate senescence (Figure 2). The timing of senescence is strictly regulated, and the genes involved in this process are called senescence-associated genes (SAGs) (Figure 2) [28]. SAGs are generally categorized as upregulated during senescence.

The source‒sink hypothesis explains senescence as a process coordinating the demands of reproductive or storage structures with supplying organs. Plant organs at a given developmental stage can be categorized as sources or sinks. Sinks import nutrients and assimilates, whereas sources supply them. These two types of organs communicate through the vascular system. Examples of sink organs in adult plants are flowers, maturing seeds, bulbs, tubers, or storage roots. Suppliers are photosynthesizing organs, and these are foliage or the green parts of inflorescence [29,30,31].

Non-dividing cells die because of the deterioration process, which results from the Second Law of Thermodynamics [29,32]. Therefore, the senescence of plant organs, which precedes the inevitable, is considered to have positive features. The genetic regulation of senescence assures efficient remobilization and reuse of macro- and micronutrients. The forwarding of nutrients to fruits and seeds increases their number and mass, which supports reproductive success. The storage organs of biennials and perennials increase their survival rate by keeping a reserve of nutrients and assimilates through adverse conditions for the next seasons of vegetative growth. Therefore, when environmental factors are favorable, they can be released and used by developing leaves or shoots, which are sink organs, as they grow.

Plants’ ability to adapt to various environmental stresses benefits from the regulation of the senescence process. Senescence can be induced in advance by many external abiotic and biotic factors, such as darkness [33,34,35,36], drought [34,37,38], ultraviolet B (UVB) exposure [39], oxidative stress [40,41], and pathogen attack [42,43,44] (Figure 2). Only mature plants respond to senescence signals, and the duration of the mature vegetative phase can be shortened under stress. Stressed plants move to the reproductive stage earlier to assure its completion at the cost of a reduced number of seeds. Hence, the induction of early senescence is a form of adaptation to a changing environment.

### 1.3. MicroRNAs Create a Complex Layer of Regulatory Modules

MicroRNAs coordinate plant development mostly by targeting mRNAs of transcription factors (TFs) [45]. MicroRNA activity is recognized in every aspect of plant life. Plant microRNAs act in response to biotic and abiotic environmental stresses [46,47,48,49]. It has been shown that a wide range of microRNAs change plant metabolism in response to drought, salinity, cold, oxidative stress, or UVB radiation. Nutrient shortage studies revealed the role of microRNAs in phosphate, sulfate, or copper homeostasis [50,51,52]. Plant hormonal homeostasis is also regulated by microRNAs [53,54,55,56,57,58,59,60]. MicroRNA-directed co-transcriptional inhibition of gene expression is their novel function. Dolata et al. (2016) showed that microRNA161 and microRNA173 genes transcription is downregulated by AGO1 under salt stress [26]. Altogether, microRNAs create multilevel and complex networks in the core of the plant regulatory processes.

In this review, we summarize the current knowledge on microRNA-regulated developmental processes leading to plant senescence. Plant competence to respond to senescence signals and to flower is also addressed. Several works have studied the microRNA roles ascribed to juvenile and mature leaf morphology and functions. MicroRNAs that play roles in vessel formation and nutrient remobilization during senescence are also included in this review. The role of microRNAs in externally induced senescence by artificial darkness or naturally occurring drought is also addressed.

## 2. Age-Related Senescence Pathways Need microRNA Action

The ability to respond to senescence signals and to transition into the reproductive stage develops only in adult plants. Indeed, it has been shown that juvenile *Arabidopsis* do not induce senescence symptoms when treated with ethylene [61]. The length of juvenile growth, however, differs widely between plant species [29]. This is particularly clear when annual and perennial plants are compared. The juvenile phase in *Arabidopsis*, for example, lasts 25 days, whereas woody plants remain in the juvenile phase for years or even decades. Once a plant matures, it enters into reproductive growth and starts to produce flowers and seeds. 

### 2.1. Sequentially Expressed microRNA156 and microRNA172 Decide between Vegetative and Generative Growth

The main regulator responsible for phase transition is microRNA156 [62]. The level of microRNA156 strongly decreases during the juvenile-to-adult phase transition. The juvenile phase can be prolonged by microRNA156 overexpression, whereas a loss-of-function mutation of microRNA156 forces plant to maturate earlier. In tobacco (*Nicotiana tabacum*), microRNA156 overexpression causes the promotion of side shoots and lateral roots development [63]. These results indicate that microRNA156 is a master regulator of vegetative growth. The second most recognized regulator of plant development is microRNA172. MicroRNA172 is expressed in succession to microRNA156 and increases in mature plants. The homeostasis between these two microRNAs regulates plant maturation and flowering [62,64,65].

Plant life expectancy differs widely between species; nevertheless, the microRNA-dependent maturation mechanism is strongly conserved in flowering species. This conservation has been shown in dicotyledonous plants, such as *Arabidopsis* [65], tomato [66,67], tobacco [68], potato [68,69], lotus [70], cabbage [71], and alfalfa [72], and in monocotyledonous maize, rice, and switchgrass [73,74,75,76]. Additionally, long-living woody species, such as apple tree (*Malus* x *domestica*) [77], tea apple (*M. hupehensis*) [78], *Populus* x *canadensis*, *Acacia confusa*, *A. colei*, *Eucalyptus globulus*, *Hedera helix*, *Quercus acutissima* [63], and gymnosperm *Sequoia sempervirens* [79], express microRNA156 to promote vegetative growth in the juvenile phase, while flowering depends on the increase of microRNA172. The sequential expression of these two microRNAs is also visible when juvenile and adult buds or leaves of an individual tree are compared.

**Figure 3:**
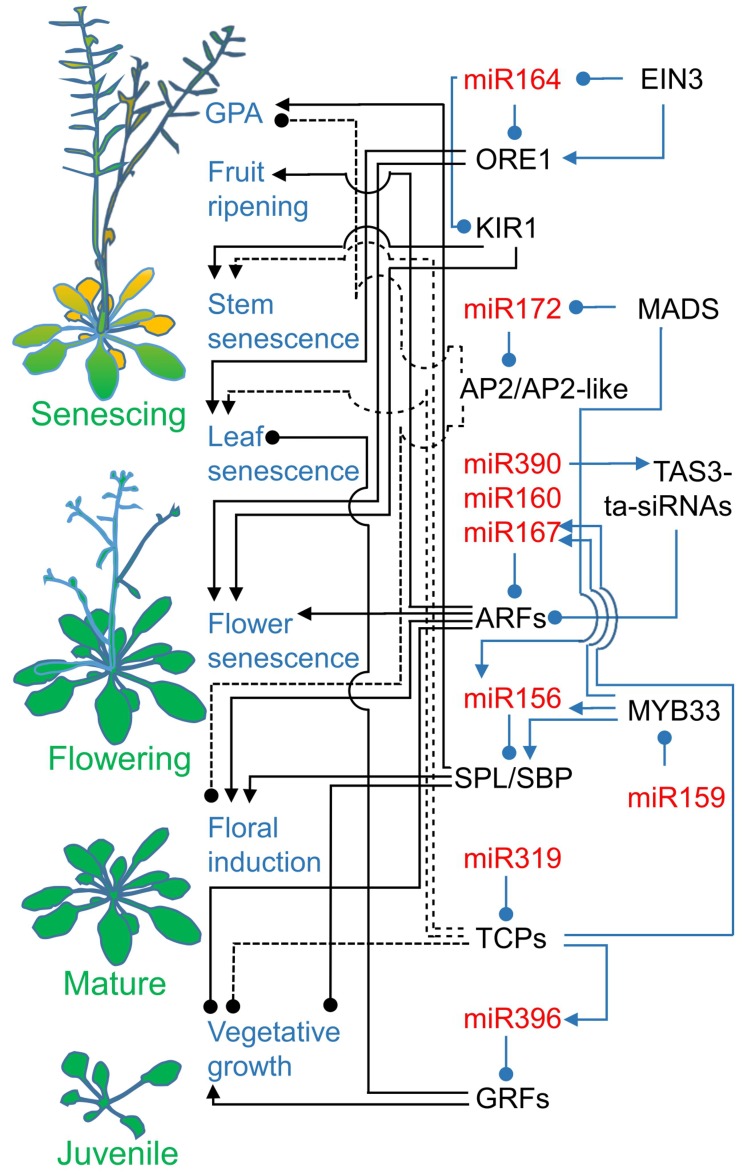
MicroRNAs’ contribution to the management of senescence in plants. MicroRNAs and their regulatory circuits are associated with various aspects of senescence. The individual panels represent plants at four developmental stages and illustrate the traits regulated by senescence-associated microRNAs. MicroRNA156 overexpression postpones flowering and promotes vegetative growth by targeting SQUAMOSA PROMOTER BINDING PROTEIN-LIKE/SQUAMOSA PROMOTER BINDING PROTEIN (SPL/SBP) transcription factors (TFs). MicroRNA172 stimulates flowering by downregulation of APETALA2 (AP2) mRNA. SPL TFs, together with microRNA172, act in the shoot apical meristem (SAM) to promote global proliferative arrest (GPA). MicroRNA160, microRNA167, and microRNA390 regulate auxin signal distribution by downregulation of AUXIN RESPONSE TFs (ARF) expression. MicroRNA396 controls leaf size and longevity by spatial and temporal restriction of growth-regulating factor (GRF) TFs expression. MicroRNA164-targeted ORESARA1 (ORE1) and KIRA1 (KIR1) stimulate flower senescence. ORE1 induces age-related cell death and de-greening in leaves. KIR1 controls cell death in stems. MicroRNA319 negatively regulates leaf senescence by targeting TEOSINTE BRANCHED/CYCLOIDEA/PCF (TCP) TFs. TCPs impact vegetative growth by inhibiting cell divisions. MicroRNAs are listed in red, proteins and siRNAs are depicted in black, regulated aspects of senescence are shown in blue, and plant developmental stages are identified in green. The arrows with triangular points reflect activation, and the arrows with circular tips reflect inhibition. The blue arrows represent regulatory circuits, and the black arrows connect circuits to regulated aspects of senescence.

MicroRNA156 downregulates SQUAMOSA PROMOTER BINDING PROTEIN-LIKE (SBP-like/ SPL)/SQUAMOSA PROMOTER BINDING PROTEIN (SBP) TFs (Figure 3). In *Arabidopsis*, microRNA156 targets the mRNAs of ten out of 16 SPLs [80]. MicroRNA156 overexpression or loss-of-function mutations of *SPLs* phenotypes reveal that SPLs negatively control the initiation rate and number of juvenile leaves, shoot branching, and adventitious root growth while the early stages of flower development are promoted. All these traits are connected to development. Gibberellic acid or floral inductive factors positively stimulate *SPLs* expression to levels higher than the microRNA156-set threshold. The microRNA156 level decreases as development progresses and the plant is competent to flower. SPL3 induces the transcription of floral meristem identity genes *LEAFY* (LFY), *APETALA1* (AP1), and *FRUITFULL* (FUL) by binding to their promoter regions. Overexpressed SPL3, SPL4, and SPL5 are capable of accelerating flowering, while their loss of function does not delay flowering. This suggests that another pathway works in parallel to the microRNA156/SPLs regulatory node [80].

MicroRNA172 is highly expressed in mature plants and acts through the translational inhibition of *APETALA2* (AP2), an A-class homeotic gene, and AP2-like targets, which include *TARGET OF EAT 1* (TOE1), *TOE2*, *TOE3*, *SCHLAFMÜTZE* (SMZ), and *SCHNARCHZAPFEN* (SNZ) (Figure 3). The overexpression of microRNA172 target proteins represses flowering, while the overexpression of microRNA172 results in an early flowering phenotype [81,82]. *AGAMOUS* (AG), a MCM1-AGAMOUS-DEFICIENS-SRF (MADS)-box TF, is a C-class homeotic gene involved in floral patterning. Its activity is involved in the determination of stamens in the third whorl of a flower and carpels in the fourth whorl of a flower [83]. A closer investigation into young floral primordia reveled that *AG* expression, limited to the center of the developing flower, overlaps with microRNA172 expression. MicroRNA172 post-transcriptionally limits *AP2* expression to the outer whorls of developing flower, where AP2 determines sepals and petals development and restricts AG function. Therefore, microRNA172 plays a dominant role in *Arabidopsis* flower patterning [84]. AP2 binds to the second intron of *AG* to act in its transcription repression [85,86]. Similarly, TOE3 binds to the second intron of *AG* to repress its expression. Moreover, *TOE3* is activated by SPL3, binding to its promoter, and in this way, *TOE3* overcomes the downregulation driven by microRNA172. Consequently, its transcript level gradually increases during development. Additionally, the SPL3–TOE3 interaction links regulatory nets of microRNA172 and microRNA156. The role of TOE3 in flower patterning is not yet established [82]. Jibran et al. (2017) proposed that AG functions not only in flower initiation, but also in flower development and senescence [87]. Binding AG to the *ANTHER DEHISCENCE 1* (DAD1) promoter induces *DAD1* gene expression to provide substrates for jasmonic acid (JA) biosynthesis. In a senescing flower, JA regulates stamen dehiscence, sepal yellowing, and perianth abscission. Such a phenomenon is postulated to be responsible for the ephemeral phenotype of *Arabidopsis* flowers [87]. Ephemeral flower senescence is independent of pollination and concurrent ethylene synthesis; thus, the flower lasts less than one day [88].

Juvenile-to-mature phase transition can be reversed during grafting. The function of microRNA156/SPL3 and microRNA172/AP2 in the process is crucial. While adult shoots of *S. sempervirens* rejuvenate when grafted onto juvenile rootstocks, the sequential expression of microRNA156 and microRNA172 is reversed [79]. Rejuvenated shoots have similar levels of the two microRNAs as juvenile shoots, as well as similar physiological characteristics such as rooting, photorespiration rates, or abscisic acid (ABA) and ethylene levels.

The developmental transition of *Physcomitrella patens* is reflected in the switching from the two-dimensional growth of a creeping protonema to the upward growth of a leafy gametophore [89]. The regulatory network of the developmental transition is only partially conserved in this gametophyte-dominated plant. MicroRNA156 downregulates SPL/SBP mRNAs, while the microRNA172/AP2 pathway is absent. Moreover, microRNA156 stimulates leafy gametophore development, promoting phase transition, which is in opposition to its function in the prolongation of the juvenile stage in angiosperms. The highest level of microRNA156 is detected during bud formation by a protonema, an intermediate stage between a protonema and a leafy gametophores. Further in development, microRNA156 is downregulated, whereas the levels of its *P. patens* targets SBP3, SBP6, and SBP13 increase [89].

The discovery that the decrease in the microRNA156 level ends the vegetative growth stage in angiosperms was a break-through in understanding the juvenile-to-adult transition. The source of the signal and mechanism initiating the time-based decrease of microRNA156 is not well known. In *Arabidopsis*, maize, and *Nicotiana benthamiana*, the signal probably derives from leaf primordia and inhibits microRNA156 expression [90]. *Arabidopsis* roots and cotyledons remain neutral during such signaling. In *Arabidopsis*, microRNA156 expression is repressed by microRNA159 overexpression, and vegetative growth is shortened [91]. Accordingly, the loss of microRNA159 results in an increase in microRNA156 and prolonged juvenile growth. MicroRNA159 decreases *MYB33* expression, the transcription factor that binds to the promoter regions of microRNA156 genes *MIR156A* and *MIR156C*, as well as to *SPL9*. The binding of MYB33 to the gene promoter regions of genes *MIR156A* and *MIR156C* and to SPL9 facilitates their expression (Figure 3). Therefore, the role of MYB33 in the maintenance of the balance between microRNA159 and microRNA156 levels is not clear [91]. MicroRNA156 and microRNA172 expression is regulated by MADS TFs. SHORT VEGETATIVE PHASE (SVP) MADS TF binds to *MIR172*’s promoter, lowering its expression [92,93]. Moreover, AGAMOUS-like 15 (AGL15) and AGL18 interact together to facilitate the expression of *MIR156* [94]. Altogether, the binding of SVP to MIR172 or the AGL15/AGL18 complex to *MIR156* promoters leads to flowering retardation. MicroRNA156 and microRNA172’s sequential expression is regulated by POLYCOMB REPRESSIVE COMPLEX1 (PRC1) components: *A. thaliana* B lymphoma Moloney murine leukemia virus insertion region1 homolog (AtBMI1-PRC1) and EMBRYONIC FLOWER (EMF1-PRC1) [95]. *MIR172* and *SPLs* are inhibited by EMF1-PRC1, which allows plants to stay in the juvenile phase. While a plant matures, *MIR156* is repressed by AtBMI1-PRC1. PRC1 components downregulate gene expression through the introduction of histone-modifying marks. Indeed, the deposition of H3K4me3 at *MIR156A* and *MIR156C* is correlated with a temporal change in the expression of microRNA156 [96,97].

### 2.2. MicroRNA160, microRNA167, and microRNA390 Guard Auxin Signaling

Auxin suppresses plant senescence by acting in many developmental pathways. In *Arabidopsis*, microRNA160 targets ARF10, ARF16, and ARF17 mRNAs from the 23 *AUXIN RESPONSE* TF genes [57], whereas microRNA167 is complementary to ARF6 and ARF8 mRNAs (Figure 3) [57,58]. MicroRNA160 levels vary in *Arabidopsis* leaves. First leaves highly express microRNA160, while in leaves that emerge later and are therefore younger, the microRNA160 expression is lower [98]. The disruption of microRNA160-driven regulation of ARF17 in *Arabidopsis* causes a number of severe developmental defects in leaf, root, and flower morphology, with the most prominent effects including reduced plant size, accelerated flowering, reduced fertility, and sometimes premature death (before flowering) [99]. MicroRNA160-ARF17 regulation is important for proper auxin signal distribution in aerial parts of the plant. In rice, *OsARF18* is expressed mainly in leaves and in spikes [100]. The deregulation of *os*-microRNA160a/b-OsARF18 modules by mutation in the microRNA binding site causes pleiotropic developmental defects. This phenotype includes dwarfing, a lower number of tillers, shorter and rolled leaves, and flower and seed abnormalities. Auxin induces the expression of *OsMIR160a* and *OsMIR160b*, as well as *OsARF18*, whereas OsARF18 represses *os*-microRNA160a/b expression in a sort of reversed feedback-loop. The importance of microRNA 160 is broader than the regulation of ARFs, because simple overexpression of ARFs has no phenotype [99].

ARF2, ARF3/ETT, and ARF4 are targeted by ta-siRNAs derived from *TAS3* non-coding RNA (TAS3-ta-siRNAs) (Figure 3) [59]. MicroRNA390 sets TAS3 phasing in TAS3-ta-siRNAs biogenesis [59,101,102]. The microRNA390-TAS3-ta-siRNAs-ARF2/3/4 regulatory module is highly conserved in land plants except for lycophytes. Normally, TAS3-ta-siRNAs delay juvenile-to-adult transition, as the neutralization of the TAS3-ta-siRNAs binding site in ARF3 or ARF4 mRNA accelerates phase change in *Arabidopsis* [103,104]. Similarly, microRNA390 overexpression delays leafy gametophore formation in *P. patens* [89]. The *Arabidopsis* phase-change phenotype is ARF3/4 dosage-dependent [103]. Nevertheless, the TAS3-ta-siRNAs, ARF3, and ARF4 RNA levels do not change during *Arabidopsis* development. Therefore, microRNA390-TAS3-ta-siRNAs-ARF3/4 probably secures a threshold below which plants do not enter the mature life stage [104]. Moreover, ARF2 is recognized as positive regulator of leaf senescence and a major player in auxin-dependent leaf longevity. Lim et al. (2010) identified an *ore14/arf2 Arabidopsis* mutant with increased sensitivity to auxin [105]. This is caused by impaired repression of auxin signaling mediated by ARF2. The mutation caused a delay in all the senescence traits tested, such as chlorophyll content, photosystem II photochemical efficiency, membrane ion leakage, and the expression of senescence-associated genes.

Flower senescence largely depends on microRNA390-TAS3-ta-siRNA-regulated ARF2. *ARF2* mRNA and proteins are accumulated during senescence. Consequently, an *arf2* T-DNA insertion mutation is characterized by the delayed yellowing of rosette leaves and the longer living plant sets flowers later and in higher number. More interestingly, the depletion of ARF2 results in delayed abscission of floral organs, and sepals remain green and turgid when abscised. Additionally, siliques dehiscence emerges later [106].

### 2.3. Temporally Acting microRNA396 and microRNA164 Regulate Leaf Longevity

An individual plant produces leaves of various shapes. Leaf morphology depends on plant age and differs between juvenile and adult plants or individual branches in the case of some woody species. In *Arabidopsis*, juvenile leaves are characterized by round, flat, and smooth blades with long petioles, whereas mature leaves have elliptical, hyponastic, and serrated blades with shorter petioles [107]. The pattern of leaf abscission is another morphological trait that differentiates juvenile and adult shoots of trees. Juvenile branches retain their leaves until spring, while adult branches drop their foliage in the fall.

Leaf growth begins on the sides of the shoot apical meristem [108,109,110]. A rod-shaped leaf primordium grows to generate flat lamina. Initially, cells proliferate throughout the primordium. Then, the cell divisions are limited to the leaf base, and the proliferation arrests. Afterwards, leaf growth is limited to the expansion of the cells [111]. Eventually, the mature leaf becomes a source of assimilates to the whole plant. Finally, the last stage of leaf development is senescence.

MicroRNA396 limits growth-regulating factors (GRF) expression (Figure 3) [112]. It targets seven out of nine *Arabidopsis* GRFs. The microRNA396 level positively correlates with the age of the leaves; it increases while leaf cells proliferate and after the proliferation arrest [113,114,115]. As a consequence, microRNA396 limits GRFs expression to the basal part of a young leaf blade containing proliferating cells, and later, it arrests GRFs activity throughout the leaf. Hence, the regulation of GRFs by microRNA396 influences the duration of leaf cells proliferation and, consequently, leaf size. Another result of microRNA396’s regulation of GRFs is the control of leaf longevity [115]. *Arabidopsis* plants expressing the modified *GRF3* gene, which is insensitive to microRNA396-driven downregulation, show not only increased leaf size, but also a late-senescing phenotype. The delayed senescence does not depend on the prolonged duration of the leaf cell proliferative phase, because the late-senescing phenotype is not present when microRNA396-insentisitve GRF3 is expressed only during the early stages of leaf development. Hence, late in development, GRFs expression influences leaf longevity but not the organ size [115].

Another temporally regulated microRNA is microRNA164. Its expression gradually decreases in *Arabidopsis* leaves during aging [116]. MicroRNA164 negatively regulates *ORESARA1 (ORE1/AtNAC2/ANAC092)* TF expression (Figure 3). ORE1 mRNA downregulation takes place in younger leaves and is released during aging due to the decline of microRNA164 [116,117]. Moreover, the increase of *ORE1* expression is directly driven by the binding of ETHYLENE INSENSITIVE3 (EIN3) to its promoter region [118,119]. EIN3 is a key TF stabilized by ETHYLENE INSENSITIVE2 (EIN2/ORE2/ORE3). *MIR164* transcription is inhibited by the direct binding of EIN3 to its promoter. The EIN2-EIN3-ORE1/MIR164-ORE1 pathway is the effector of a signaling cascade of ethylene, a hormone known to accelerate senescence. ORE1 is a positive regulator of senescence and age-related cell death in *Arabidopsis* leaves. *ore1* mutants’ late-senescence phenotype is characterized by delayed cell death and, therefore, the loss of chlorophyll content, loss of photochemical efficiency (Fv/Fm), increase in membrane ion leakage, and increased cysteine protease-encoding *senescence-associated gene 12* (SAG12) expression [120]. Moreover, *ein3* mutants are characterized by a stay-green phenotype. Conversely, cell death occurs earlier during leaf development in *microRNA164a/b/c* mutants. The *Arabidopsis* ethylene-induced senescence signaling pathway includes several members of NAM/ATAF1,2/CUC2 (NAC) TFs [118,119]. Except *ORE1*, EIN3 induces *NAC-LIKE ACTIVATED BY APETALA3/PISTILLATA* (AtNAP/ANAC029) expression, whereas EIN2 promotes the expression of *ANAC019*, *ANAC047*, *ANAC055*, and *ORS1/ANAC059*. EIN3-induced ORE1 and AtNAP activities have partially additive functions in age-dependent and artificially induced leaf senescence. ORE1 induces *ANAC087* and *ANAC102* expression, while ORE1 and AtNAP activate three common NAC TF genes *ANAC041*, *ANAC079*, and *VND-INTERACTING2* (VNI2). However, VNI2 is known as a negative regulator of leaf senescence [121]. The wide cascade of ethylene-induced NAC TFs assures elasticity in promoting senescence. Notably, microRNA164-regulated ORE1 is directly involved in leaf de-greening during senescence. ORE1 binds to the promoter regions of *STAY-GREEN1* (SGR1/NYE1), chlorophyll *b* reductase (*NYC1/NOL*), and pheophorbide a oxygenase (*PaO*)—proteins essential in chlorophyll catabolism—to facilitate their expression [120]. In the case of NYE1 and NYC1, ORE1 function is additive to EIN3, which also binds to the promoters of the genes encoding the same proteins but in a separate promoter site. Moreover, ORE1 stimulates ethylene biosynthesis in a feed-forward manner by inducing 1-aminocyclopropane-1-carboxylicacid synthase (ACS2).

### 2.4. Cell Divisions and JA Biosynthesis Are Linked to Leaf Senescence by microRNA319 Regulatory Modules

MicroRNA319 regulates five TEOSINTE BRANCHED/CYCLOIDEA/PCF TFs (TCPs) known as negative regulators of cell differentiation and positive regulators of senescence (Figure 3) [3]. The function of miRNA319 (miR-JAW) as a negative regulator of plant senescence was first shown in a microRNA319a-overexpressing *Arabidopsis* mutant (*jaw-D*). This mutant shows the delayed leaf senescence/prolonged juvenile-phase phenotype that is reversed by the addition of JA [122]. Mild overexpression of microRNA319-non-targeted TCP4 in *jaw-D* also relieves the phenotype [123]. Schommer et al. (2008) linked the microRNA319-TCP regulatory module to the JA biosynthesis pathway [122]. TCP4 induces *LIPOXYGENASE2* (LOX2) expression, encoding a chloroplast-localized enzyme dedicated to the JA biosynthesis pathway [122,124,125]. The TCP4 binding motif in the promoter region of *LOX2* links this pathway to development, as TCP4 does not react to environmental stimuli. A consequence of this is a higher JA biosynthesis rate in response to developmental changes. JA is a positive regulator of senescence. Nevertheless, JA on its own is not essential for senescence to occur, as JA biosynthesis (*aos, opr3, LOX2-RNAi*) or JA-signal transduction defective mutants (*coronatine insensitive1, coi1*) are not impaired in senescence. Hence, the endogenous JA level is not a senescence-limiting factor. Exogenously applied JA accelerates senescence in *jawD*, as well as wild type (WT) plants, while JA-signal transduction defective mutant *coi1* stays insensitive to JA. It cannot be ruled out, however, that JA promotes senescence as an exogenous signal and that, therefore, it is a way of communicating between plants to coordinate developmental processes. The role of type II TCPs in cell proliferation arrest is better understood. TCP4 promotes the expression of *CYCLIN-DEPENDENT KINASE INHIBITOR 1 (ICK1)/KIP RELATED PROTEIN1* (KRP1) working in a pathway inhibiting the progression of the cell cycle [126]. MicroRNA319-regulated TCP4 directly induces the expression of *MIR396b* in *Arabidopsis*. The binding of TCP4 to the *MIR396* promoter results in differentiated expressions of microRNA396 along the leaf with the highest level at the distal parts of the organ. The accumulation of this microRNA increases with leaf age. The role of the expressed microRNA396b is to withhold cell proliferation by targeting GRF TFs [126,127].

### 2.5. MicroRNA164 Contributes to Flower and Shoot Cell Death

Transcription factor ORE1, which also influences leaf longevity, and KIRA1/ANAC074 (KIR1) induce programmed cell death in stigma [128]. They are highly transcribed in senescing stigma; consequently, the lack of these two TFs delays stigma senescence. Moreover, the TFs transiently expressed in tobacco leaf or ectopically expressed in *Arabidopsis* cause leaf yellowing and extensive cell death, respectively. The *Sorghum bicolor* orthologue of KIR1 (D) induces programmed cell death in pitch parenchyma of stalks, creating air-filled spaces [129]. The same functions of KIR1 were reported in *Arabidopsis* shoots. Among other functions, the formation of empty spaces in stalks facilitates nutrient remobilization from source to sink tissues. Moreover, it is responsible for the dry stem trait in sorghum, while cultivars with juicy stalks have nonfunctional alleles of *D*. The KIR1 transcription factor is conserved among flowering plants and is a master transcription factor inducing programmed cell death [129]. AtORE1 is known to be post-transcriptionally regulated by microRNA164; nevertheless, the dependency of ORE1 activity on microRNA164 is not known in flower organs or inflorescence stems. KIR1, the most recently identified NAC TF, possesses the recognition site for microRNA164.

### 2.6. MicroRNA319-Regulated TCP4 Promotes the Remobilization of Resources

Floral development and seed setting is linked to increased and accelerated demand on nutrients and assimilates, which are supplied by leaves. Intensive vessel formation can also be observed in senescing plants [123]. This may be caused by the physical distance between the flowers or developing fruits and organs providing nutrients and assimilates. Accelerated vessel formation is linked to the intensification of secondary cell wall biosynthesis and the initiation of programmed cell death. This is caused by the induction of *VASCULAR RELATED NAC-DOMAIN PROTEIN 7* (VND7), a NAC-family TF, activating the transcriptional network leading to xylem vessel formation [123]. *VND7* is induced by the direct binding of the TCP4 transcription factor to the *VND7* promoter region. The TCP4 mRNA is targeted for cleavage by microRNA319 (Figure 3), and the level of the latter declines while the plant matures [130,131]. Therefore, *TCP4* expression and, consequently, *VND7* expression gradually increase during plant development. Micro319-resistant *TCP4* overexpression leads to VND7 overexpression, which further activates many secondary cell wall forming genes, such as cellulose synthases *CesA4* and *CesA8*, *ARABIDOPSIS PHENYL AMMONIA LYASE1* (PAL1), *CINNAMATE-4-HYDROXYLASE* (C4H), *COUMARATE 3-HYDROXYLASE* (C3H1), and laccases *Lac4* and *Lac17* [123].

### 2.7. MicroRNAs in Fruit Ripening and Senescence: Agronomical Traits

Senescence, as a process, according to the source‒sink hypothesis, redirects nutrients and assimilates from leaves, which have fulfilled their role as a source, to developing fruits. The involvement of microRNAs in the mobilization of resources is made obvious by the different and often opposite expression trends of many microRNAs when senescing *Arabidopsis* leaves and siliques have been compared [117]. Therefore, the manipulation of leaf senescence can be used as a tool to improve the yield, quality, or shelf life of fruits and grains. Tomato, *Solanum lycopersicum*, is a fleshy, climacteric fruit model plant, as well as an economically important crop plant. Indeed, knocking down the expression of *SlORE1S02*, an *AtORE1* ortholog with a disrupted microRNA164 hybridization site, led to delayed senescence, which was evidenced by a stay-green phenotype [132]. Moreover, as a result, the fruit yield was higher and had an increased soluble sugar content.

Tomato fruit coloration largely depends on microRNA156 expression. The level of microRNA156 decreases during tomato ripening, and the overexpression of microRNA156 results in the pale red coloration of ripened fruit [133,134]. A natural epigenetic mutation in *COLORLESS NON-RIPENING* (CNR) of tomato, a SBP-box TF gene whose mRNA is targeted by microRNA156, has been identified. *CNR* is upregulated in fruit during the breaker stage, consequently, red coloration in the fruits of the CNR mutants is absent. Other important targets for tomato ripening and softening include endo-1, 4-beta-glucanase, pectate lyase, and beta-galactosidase, which are targeted by microRNA396, microRNA482, and novel microRNAZ7, respectively [134]. Importantly, these three microRNAs are upregulated during the breaker stage of tomato. In contrast, ethylene treatment, which is responsible for the red coloration of climacteric fruits, decreases the expression of these microRNAs and microRNA156. Microtranscriptome analysis reveals a global decrease of known conserved and known non-conserved microRNAs during tomato fruit ripening from the mature green to the red stage [134]. In *Solanaceae*, microRNA1917 is involved in pedicel abscission [135]. MicroRNA1917 targets tomato *CONSTITUTIVE TRIPLE RESPONSE1-LIKE4* (CTR4) splicing variant 3 (SlCTR4sv3), which is specifically expressed in the abscission zone. *SlCTR4* regulates ethylene signaling. Sly-miR1917 overexpression changes tissue-specific ethylene responses due to increased ethylene synthesis. In adult plants, this results in accelerated pedicel abscission and fruit maturation. In other plants, CTR expression is induced in ripening fruits and in cut flowers during storage, but the microRNA1917-regulatory module is not conserved out of *Solanaceae*.

MicroRNAs known to regulate *Arabidopsis* development and senescence were identified in non-climacteric fruits during ripening or postharvest senescence. Strawberry *Fragaria ananassa* is a model plant for non-climacteric fruits. The edible part of a strawberry is the receptacle. Initially, it grows under the hormonal control of achenes [136]. Later, the color change is driven by the action of several TFs. The source-to-sink transition during the color change of the receptacle is driven by the gibberellin- and abscisic acid-regulated MYB family TF (GAMYB). GAMYB binds to the promoter regions of GA-responsive genes to activate their expression. In strawberry, *FaGAMYB* knockdown results in retained photosynthetic activity accompanied by a decrease in anthocyanin and sucrose concentration [137,138,139]. The role of *Fa*GAMYB in the red coloration of the strawberry receptacle can be linked to *FaMYB10* and *FaMYB1* expression induction, as MYB TFs promote anthocyanin biosynthesis. Generally, the downregulation of FaGAMYB results in the changed expression of 2624 genes; therefore, its role can be considered to be dominant during the ripening of the strawberry receptacle. The role of microRNA159 in the process has been well described [137,140]. A high concentration of gibberellic acid downregulates *fan*-miR159a, one of the two microRNA159 that target GAMYB. Low levels of the two microRNA159 overlap with the peak of GAMYB expression. In strawberry, high-throughput sequencing of small RNAs revealed several microRNAs and their targets, which act during the postharvest senescence of the receptacles [141]. These microRNAs are microRNA156, microRNA160, microRNA164, microRNA167, microRNA172, and microRNA390. A comparative analysis of microRNAs and their targets in fruits was also conducted for the spontaneous, late-ripening mutant of sweet orange *Citrus sinensis* Fengwan and wild-type Fengjie 72-1 [142]. From the whole pool of conserved microRNAs, only 15% were differentially expressed between the two cultivars. As expected, among them were microRNA156 and microRNA159. Therefore, the SPLs and GAMYBs pathways are differentially regulated between these two cultivars. Interestingly, 38% of the novel citrus microRNAs were deregulated between the differently ripening cultivars. This suggests that species-specific microRNAs are responsible for observed cultivar variations, which opens new pathways for agronomically important research. Unusual targets of conserved microRNAs with important roles in fruit storage have also been identified. The pink-to-brown change of the peel coloration of *Litchi* fruit is a commercially recognized, senescence-associated trait [143]. Important for *Litchi* fruit storage, the positive regulators of the anthocyanin biosynthesis pathway are MYB TFs [138,139]. In *Litchi*, one of the MYB TFs is targeted by microRNA858. The expression of microRNA858 increases during postharvest senescence in *Litchi*, which may negatively influence the pink coloration of the peel. The flavonoid biosynthesis enzyme chalcone isomerase is regulated by microRNA396 in *Litchi*. MicroRNA396 is upregulated during room temperature storage, while cold room temperatures decrease its expression. Another interesting target of *Litchi* microRNA396 is the mRNA of the proteolytic enzyme CYSTEINE PROTEASE1 CP1. Proteolysis leads to cell death and therefore is an important part of the late senescence of *Litchi* fruit. The CP1 mRNA level fits to the expression pattern of its regulatory microRNA. These targets have not been previously reported; nevertheless, such additional regulatory nodes can be a part of species-specific posttranscriptional regulation [143].

### 2.8. MicroRNA156 and microRNA172, the Main Drivers of Plant Development, Also Act in Global Proliferative Arrest

Grains, legumes, and other economically important plants, as well as *Arabidopsis*, are characterized by a single reproductive cycle, semelparity, after which the plant dies. This is due to synchronized arrest of the meristem cell divisions known as global proliferative arrest (GPA). GPA is followed by grain filling and occurs after a given number of fruits is set. Therefore, GPA is assumed to target the allocation of resources to growing seeds. Age-control of shoot apical meristem (SAM) divisions by GPA is also regulated by microRNAs (Figure 3). Balanza et al. (2018) identified *ful-1* (Ler) and *ful-2* (Col) mutants with prolonged activity of SAM and, consequently, greatly delayed GPA [144]. Interestingly, the elimination of the microRNA172 binding site in AP2 enhances the phenotype of the *FUL* defective mutant in that the GPA is prevented and the meristem stays active. The expression of AP2 that cannot be regulated by microRNA172 in senescent wild-type plants that have undergone GPA restores SAM activity, which makes new flowers and fruits. *FUL* and microRNA172 are overexpressed during inflorescence development to inhibit *AP2* and *AP2-like* expression. At the same time, the microRNA156 level drops, and upregulated SPL factors strongly induce *FUL* [144]. AP2 is a positive regulator of the *WUSCHEL* (WUS) gene, a homeodomain transcription factor maintaining an active stem-cell pool [145]. Therefore, it is proposed that this mode of regulation has a general role in setting perennial flowering and in switching from monocarpic to polycarpic habits [144].

## 3. Induced Senescence Pathways Involve microRNAs

A plant’s reaction to drought is ABA synthesis to induce short- and long-term responses to stress [146]. One of the long-term responses is drought-induced senescence. Drought causes leaf senescence, which is visible as yellowing. In contrast, stay-green phenotypes are associated with reduced senescence symptoms in drought-treated mature plants. There are number of mutations causing the stay-green phenotype and elevated drought resistance as an additional trait. One way of stress- or senescence-induced chloroplast degradation is the formation of chloroplast vesicles. In *Arabidopsis*, the process depends on *CV* (for chloroplast vesiculation) gene expression. *CV* silencing delays senescence symptoms and increases drought tolerance [147]. Moreover, in drought-treated stay-green quantitative trait locus sorghum plants, senescence occurs later [148]. Similarly, in tobacco, overcoming a rate-limiting step in cytokinin synthesis through isopentenyltransferase expression in senescing plants resulted not only in a stay-green phenotype, but also in extreme drought resistance [149]. Thus, microRNAs, as molecules regulating a wide range of plant responses, are also linked to drought-induced senescence.

### 3.1. MicroRNA165/166, microRNA164, and microRNA393 are involved in Senescence Accelerated by Drought

**Figure 4:**
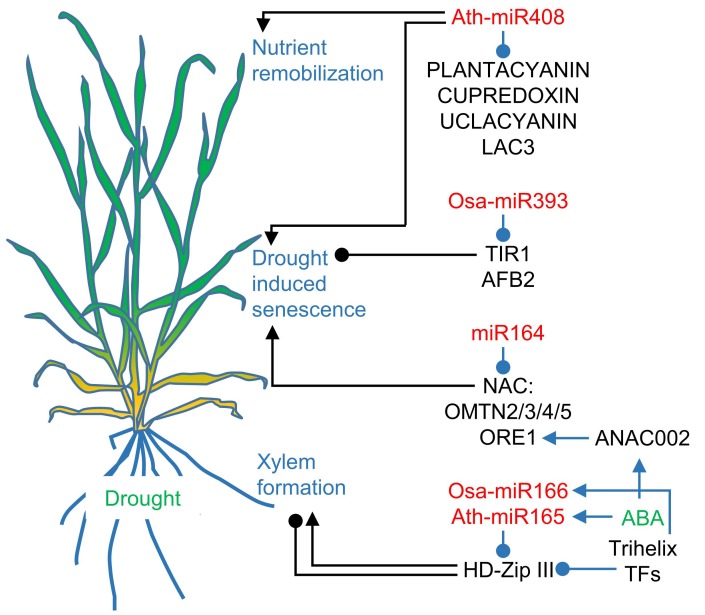
MicroRNAs regulate drought-induced senescence. The panel shows drought- and senescence-related circuits involving microRNAs. The induction of microRNA165/166 alters xylem formation by modulation of class III homeodomain leucine zipper (HD-Zip III) abundance in the vasculature. MicroRNA164 downregulates NAM/ATAF1,2/CUC2 (NAC) TFs, which promote drought-induced senescence. Additionally, abscisic acid (ABA) induces ORESARA1 (ORE1), a senescence-associated TF. MicroRNA393 targets TRANSPORT INHIBITOR RESPONSE 1 (TIR1) and AUXIN SIGNALLING F-BOX PROTEIN 2 (AFB2), which lowers auxin signaling and promotes senescence and drought sensitivity. Plant senescence or increased abiotic sensitivity coincides with the overexpression of microRNA408. MicroRNA408 targets copper-binding proteins. The microRNAs are listed in red, proteins in black, regulated traits in blue, and environmental stress in green. The arrows with triangular points reflect activation, and the arrows with circular tips reflect inhibition. The blue arrows are for regulatory circuits, and the black arrows connect circuits to regulated traits.

Class III homeodomain leucine zipper (HD-Zip III) TFs mRNAs levels are controlled by microRNA165/166 (Figure 4) [57]. HD-Zip III TFs determine xylem cell types [150]. Higher levels of HD-Zip III induce central metaxylem formation, while lower levels of HD-Zip III induce side protoxylem formation. MicroRNA165/166 expressed in the endodermis around the vasculature determine the HD-Zip III gradient in neighboring tissues. Drought treatment of rice seedlings induces trihelix TFs, which inhibits the expression of OsHB3, a rice Class III HD-Zip TF, and simultaneously induces *OsMIR166i*. OsHB3 is targeted by the induced osa-miR166i-3p [150]. The OsHB3 function in senescence can be attributed to its role in vascular patterning. A similar function can be attributed to microRNA166-targeted OsHB5, which is specifically expressed in phloem, but the disruption of the microRNA166/OsHB5 regulatory node results in an altered xylem formation [151]. In *Arabidopsis* seedling roots, exogenous ABA is needed to develop continuous xylem strands [152]. ABA or drought treatment activate the *MIR165A* gene in the endodermis. As a consequence, HD-ZIP III TFs in the stele are inhibited, which results in the development of extra xylem strands.

Rice overexpressing Oryza miR164-targeted NAC2 (OMTN2), OMTN3, OMTN4, and OMTN5 TFs develops sensitivity to drought, which is visible in wilting and leaf rolling [153]. Additionally, the fertility of the plants is reduced in comparison to WT after severe drought treatment. The expression levels of the four rice NAC TFs is regulated by microRNA164 (Figure 4). The direct involvement of drought stress in NAC TFs regulated pathways requires the presence of ABA. In *Arabidopsis*, ABA synthesis induces the expression of *ARABIDOPSIS THALIANA ACTIVATING FACTOR1/ARABIDOPSIS NAC002* (ATAF1/ANAC002) and further, ATAF1/ANAC002 induces *ORE1* expression, a positive regulator of senescence (Figure 4) [154]. It has been observed that impairment in ABA signaling, such as in ABA-insensitive *abi1* and *abi2* mutants, delays leaf senescence [155]. NAC TFs are strongly involved in senescence regulation, and it seems that this regulation involves drought responses. Therefore, it would be interesting to study microRNA involvement in NAC TFs expression regulation, because these proteins represent a potential target for obtaining drought-tolerant plants.

MicroRNA393 regulates plant responses to environmental stimuli. It also influences flowering time, leaf morphogenesis, nutrient homeostasis, and root architecture [156,157]. Hyposensitivity to auxins in *OsMIR393*-overexpressing mutants result in enhanced tillering, early flowering, and reduced tolerance to drought [158]. The accelerated development of rice overexpressing microRNA393 coincided with enhanced drought sensitivity, which is in line with observations that delayed-senescence phenotypes are often drought resistant. This is likely because the higher level of microRNA393 lowers auxin signaling by targeting TRANSPORT INHIBITOR RESPONSE 1 (TIR1) and AUXIN SIGNALLING F-BOX PROTEIN 2 (AFB2), which are auxin receptors (Figure 4) [60].

### 3.2. Drought and Darkness Uncover microRNA408 as a Senescence-Associated microRNA

In *Arabidopsis*, microRNA408 accumulates naturally in the very late stages of normal senescence, as well as in dark-induced senescence [159]. However, the overexpression or inactivation of *MIR408* has no effect on natural senescence. Instead, the accumulation of microRNA408 leads to more vigorous growth as individual leaves and seeds become larger. In contrast, low levels of microRNA408 have more significant consequences in smaller plants. Abiotic stress treatment revealed an early senescence phenotype of *Arabidopsis* that overexpresses *MIR408*, while lower levels of microRNA408 delayed senescence (Figure 4). Moreover, the downregulated expression of *MIR408* resulted in better tolerance to drought and osmotic stress than WT, while overexpressed microRNA408 resulted in plants exhibiting lower tolerance to both stresses. Drought and osmotic stress slightly decrease the microRNA408 level. Similar to the effects in *Arabidopsis*, microRNA408 in pea was downregulated during drought [160]. The drought treatment of other species, such as *Medicago truncatula*, upregulated microRNA408 [161]. Moreover, the overexpression of microRNA408 in rice resulted in the drought tolerance of specific lines [162]. Interestingly, *Arabidopsis* plants overexpressing *MIR408* were more tolerant to other abiotic stresses, such as salinity, cold, and oxidative stress than plants with knocked-down microRNA408 expression and WT plants. *Arabidopsis* microRNA408 targets mRNAs coding for the following copper-binding proteins: PLANTACYANIN, CUPREDOXIN, UCLACYANIN, and LACCASE (LAC3). The first three are electron transfer proteins, while the last one catalyzes the oxidative polymerization of lignin. In naturally senescing *Arabidopsis* leaves, the targets’ expression are not related to the microRNA408 level. *PLANTACYANIN* and *UCLACYANIN* expression increases in late senescence, LAC3 mRNA levels are stable throughout development, and only *CUPREDOXIN* displays higher expression in juvenile leaves when compared with mature and senescing leaves [159]. Basically, microRNA408 targets negatively correlate with microRNA408-induced expression during copper deficiency stress [163]. Importantly, microRNA408 plays a central role in the copper and light signaling driven by SPL7 and ELONGATED HYPOCOTYL5 (HY5), respectively. SPL7 and HY5 interact together to induce *MIR408* expression. Moreover, constitutive expression of microRNA408 rescues the *spl7*, *hy5*, and *spl7hy5* phenotypes [164]. The involvement of microRNA408 in senescence is not well understood; however, its function may be related to nutrient remobilization.

### 3.3. Light Conditions Alter microRNA Levels

Prolonged darkness results in leaves yellowing, resembling senescence characteristics. It is not known whether artificially induced senescence at the level of microRNAs is similar to naturally occurring senescence. High reactive oxygen species (ROS) production is characteristic to darkness-treated plants. MicroRNA398 is crucial for ROS tolerance, as it targets cytosolic Cu/Zn superoxide dismutase (CSD1) and chloroplastic Cu/Zn superoxide dismutases (CSD2). *Arabidopsis* overexpressing *CSD2* that is resistant to microRNA398 is highly tolerant to oxidative stresses, such as, for example, during high-light exposure; nevertheless, the tolerance level of the mutant to prolonged darkness is not known [165]. Microarray study of microRNA expression was conducted in darkness-treated four-week-old *Arabidopsis*. The most profound changes were observed for 44 stress-induced microRNAs. The senescence-associated microRNAs induced were microRNA156, microRNA160, microRNA172, microRNA396, microRNA166, and microRNA408, while the downregulated microRNAs were microRNA159, microRNA390, microRNA164, and microRNA167 [166]. Importantly, general studies concerning microRNA biogenesis in darkness have been recently conducted. Under day-light conditions, a cytoplasmic pool of HYL1 is protected from degradation indirectly by Constitutive photomorphogenic 1 (COP1), while at night, COP1 migrates to the nucleus and HYL1 is prone to degradation [167]. HYL1 phosphorylation prevents night-induced HYL1 degradation. During unusual periods of prolonged darkness/shading, a pool of nuclear HYL1 is phosphorylated and stays in an inactive state, which inhibits the night-induced HYL1 degradation mechanism. Upon light exposure, the phosphorylated HYL1 is dephosphorylated to restore microRNA biogenesis [168]. MicroRNA production is impaired under continuous high-light conditions in the barley WHIRLY1 knockdown mutant. WHIRLY1 is a DNA/RNA binding protein, but the exact function of WHIRLY1 in the microRNAs biogenesis is not known [169].

## 4. Age Influences the Efficiency of microRNA-Driven Posttranscriptional Gene Expression Regulation

Plant aging may influence microRNA biogenesis or the overall efficiency of microRNA-driven posttranscriptional gene expression regulation. As mentioned above, microRNAs modulate gene expression at the posttranscriptional level by targeting mRNAs to cleavage or translation inhibition [1,4,5]. The changes in the contributions of both processes to microRNA-driven actions during plant development are shown in *Arabidopsis* modified to combine the expression of artificial microRNA and its Firefly luciferase target (LUC) [170]. The ratio between the LUC protein and its mRNA levels increases with age, suggesting that the contribution of artificial microRNA-driven co-translational inhibition of LUC expression is reduced. Moreover, the accumulation of the artificial microRNA declines during *Arabidopsis* development, but this is not correlated with a failure of microRNA biogenesis machinery expression, indicating the increasing role of the RNA degradation processes activated during senescence [170,171]. Such results suggest that the overall dynamics of microRNA contribution to gene expression regulation may change during plant development. The pathways responsible for wide, age-dependent microRNA expression regulation are not known. Interestingly, in maturing barley plants, the biosynthesis of 20 nt long 5’ U-miR156-5p is shifted towards 21 nt long 5’ UU-miR156-5p [172]. Both molecules originate from the same gene. This observation may reflect an age-dependent deregulation of the precision of microRNA maturation. The experimental analysis of the barley microRNA156-5p target revealed that the 5’ UU-miR156-5p isoform is not able to guide the cleavage of two barley SPL TFs out of the six SPLs targeted by the 20 nt long 5’ U-miR156-5p. It is not known whether such mechanism of age-dependent microRNA action inhibition can be applied to a wider set microRNAs. It would be very interesting to study the mechanisms leading to various isomiRs’ production during the life of the plant [173].

## 5. Conclusions

MicroRNAs reveal another layer to the regulation of plant life processes. Many microRNA-driven, senescence- and development-associated characteristics are recognized as putatively useful in fruit and grain production. The most thoroughly recognized, senescence-related microRNAs are temporally regulated. While general factors regulating microRNA biogenesis and *MIR* expression have been studied intensively, the induction of temporal changes guarded by microRNAs is still poorly understood. Hunter et al. (2006) suggested that microRNA-dependent ta-siRNAs can guard the uniformity of a plant organism entering the adult stage of development [104]. Ta-siRNAs are not inducers of the juvenile-to-adult phase transition, as their expression is stable during the plant’s life. Their putative role is to coordinate simultaneous development in various tissues as they are transmitted over long distances in a plant. According to the source‒sink hypothesis, in various plant organs, different responses to senescence signals are displayed. Spatial modulation of senescence characteristics can be achieved by individually regulated *MIRs*. Large gene families of some microRNAs are interpreted as being differentially expressed within plant organs or tissues. Therefore, microRNA-driven transcriptional and posttranscriptional gene expression regulation is a way to synchronize and control various developmental and senescence processes in plants. Processes such as splicing efficiency and alternative splicing of pri-miRNAs, alternative polyA site selection in pri-miRNAs, the role of CHR2 helicase in the stem-loop melting process, and the equilibrium of alternative structures of stem-loops in pri-miRNAs in age-related or stress-related conditions should be investigated in greater depth in the future [173,174,175,176,177]. These processes are still poorly understood in senescing plants and require further experiments to be elucidated.
